# Psychosocial risk factors in home and community settings and their associations with population health and health inequalities: A systematic meta-review

**DOI:** 10.1186/1471-2458-8-239

**Published:** 2008-07-16

**Authors:** Matt Egan, Carol Tannahill, Mark Petticrew, Sian Thomas

**Affiliations:** 1 Medical Research Council Social and Public Health Sciences Unit, 4 Lilybank Gardens, Glasgow, G128RZ, UK; 2 Glasgow Centre for Population Health, Level 6, 39 St Vincent Place Glasgow, G12ER, UK; 3 Public and Environmental Health Research Unit, London School of Hygiene and Tropical Medicine, Keppel St., London WC1E 7HT, UK; 4 Medical Research Council Social and Public Health Sciences Unit, 4 Lilybank Gardens, Glasgow, G128RZ, UK

## Abstract

**Background:**

The effects of psychosocial risk factors on population health and health inequalities has featured prominently in epidemiological research literature as well as public health policy strategies. We have conducted a meta-review (a review of reviews) exploring how psychosocial factors may relate to population health in home and community settings.

**Methods:**

Systematic review (QUORUM) of literature reviews (published in any language or country) on the health associations of psychosocial risk factors in community settings. The literature search included electronic and manual searches. Two reviewers appraised included reviews using criteria for assessing systematic reviews. Data from the more robust reviews were extracted, tabulated and synthesised.

**Results:**

Thirty-one reviews met our inclusion criteria. These explored a variety of psychosocial factors including social support and networks, social capital, social cohesion, collective efficacy, participation in local organisations – and less favourable psychosocial risk factors such as demands, exposure to community violence or anti-social behaviour, exposure to discrimination, and stress related to acculturation to western society. Most of the reviews focused on associations between social networks/support and physical or mental health. We identified some evidence of favourable psychosocial environments associated with better health. Reviews also found evidence of unfavourable psychosocial risk factors linked to poorer health, particularly among socially disadvantaged groups. However, the more robust reviews each identified studies with inconclusive findings, as well as studies finding evidence of associations. We also identified some evidence of apparently favourable psychosocial risk factors associated with poorer health.

**Conclusion:**

From the review literature we have synthesised, where associations have been identified, they generally support the view that favourable psychosocial environments go hand in hand with better health. Poor psychosocial environments may be health damaging and contribute to health inequalities. The evidence that underpins our understanding of these associations is of variable quality and consistency. Future research should seek to improve this evidence base, with more longitudinal analysis (and intervention evaluations) of the effects of apparently under-researched psychosocial factors such as control and participation within communities. Future policy interventions relevant to this field should be developed in partnership with researchers to enable a better understanding of psychosocial mechanisms and the effects of psychosocial interventions.

## Background

In recent years, the effects of psychosocial risk factors on population health have received considerable attention in both research and policy circles [1-5]. Psychosocial epidemiology explores the way peoples' interactions with their social environments may influence health either directly (e.g. through biological responses to what is commonly called 'stress') or indirectly through health behaviours [5]. This research is controversial (for example, some researchers contest the evidence that psychosocial factors are important health determinants, particularly compared to material/economic determinants of population health [6]). Nonetheless, psychosocial theories are influential and have encouraged policy-makers to develop public health strategies that consider people's support networks, sense of control and empowerment, their sense of security, and the extent to which people participate in the local community and civic society [2].

A range of different psychosocial risk factors have been posited as potential contributors to ill health and health inequalities. The evidence that describes these risk factors – the places in which they occur, the kinds of people they effect, and the health effects that they are associated with – could suggest points of intervention and facilitate the targeting of resources more effectively on measures that will reduce ill health and its causes in the community [7-9]. Literature reviews have synthesised epidemiological evidence describing associations between various psychosocial factors and population health characteristics in different social settings [3,4,10-13]. To collate evidence for researchers and practitioners interested in community health we have conducted a meta-review (a review of reviews) exploring how psychosocial factors may relate to population health in community settings.

### Defining 'psychosocial'

A major obstacle to reviewing this area is the lack of consensus regarding the definitions and usage of psychosocial concepts in the research literature. Martikainen et al suggest that psychosocial factors most usefully describe a bridging or 'meso-level' between individual and social structures and hence include such factors as support from social networks, control at work or in the home, effort/reward imbalance, security and autonomy, and work-family conflict [14]. They state that a psychosocial explanation of health should describe how macro- and meso-level social processes lead to perceptions and psychological processes at the individual level. We have made this bridging role a central component of our own definition of psychosocial: seeking to exclude macro-level risk factors that are unlikely to effect health via psychological processes, and exclude psychological characteristics (e.g. depression, anxiety and type 'A' characteristics) that described individuals rather than some form of interaction between people and their social environment involving psychological processes.

In practice, identifying psychosocial characteristics can be a difficult task, the results of which may be contestable. Martikainen et al highlight one difficulty when they argue that psychosocial exposures do not necessarily invoke psychosocial processes, and may arrive at health outcomes through alternative (non-psychosocial) pathways. For example, social networks provide instrumental and material benefits as well as emotional support from friends and family; yet they consider only the latter path to qualify as a psychosocial process [14].

A recent meta-review of non-health sector psychosocial interventions (conducted by the present authors) corroborated the view that psychosocial terminology is frequently employed without consensus or definition [Egan M, Thomson H, Petticrew M, Tannahill C, Kearns A, Hanlon P: What are 'psychosocial interventions' and how might they improve health?, submitted]. It also found that at the level of systematic review, most of the available evidence on the health impacts of community-based psychosocial interventions comes from studies of workplace psychosocial interventions. The review concluded by calling for better theory to guide research in population health and social epidemiology (others have made similar calls [15]). In terms of underlying theory, research into psychosocial factors in workplace settings tends be framed around two well-known theoretical models of the workplace psychosocial environment (i.e. the 'demand control support model' and the 'effort-reward imbalance model') [3,4,10]. In contrast, we have found research into health and wellbeing in the wider community to be open to a broader, but less consistently described, array of theoretical concepts that do not always clearly distinguish psychosocial and non-psychosocial components [16-19].

One of the most influential theoretical frameworks derives from Putnam's work on social capital, which he defines as 'features of social organisations, such as networks, norms, and trust, that facilitate action and co-operation for mutual benefit' [16]. Key to this approach is the hypothesis that strong social interactions between residents of a neighbourhood can benefit not only those who interact (e.g. in terms of emotional, practical and financial forms of social support), but also to neighbours who do not take part in these interactions. These latter benefits are referred to as 'externalities' and may include increased feelings of safety, reductions in anti-social behaviour, and better services and amenities in neighbourhoods where communities are cohesive enough to give individuals and groups the confidence to engage in informal social control (e.g. intervening to prevent anti-social behaviour) and civic participation (e.g. establishing youth groups, participating in local decision-making, intervening to improve the local area, etc).

Referring to Martikainen et al [14], we would argue that some components of social capital can be considered psychosocial factors because they are likely to involve social processes that lead to perceptions and psychological processes at the individual level (e.g. community characteristics that encourage mutual trust, emotional support and participation/control). However, social capital also includes components that may be associated with 'non-psychosocial' pathways to health, such as practical support and improved local services, and with macro-level contextual factors which impact on health above and beyond psychosocial effects.

In their work on social cohesion, Stafford et al have developed a measurement tool that focuses on 8 components of neighbourhood social cohesion: (1) family ties (frequency of contact with local family); (2) friendship ties (frequency of contact with local friends); (3) participation (regular participation in local organised groups, such as social, religious, neighbourhood interest, evening classes, etc); (4) integration into wider society (contact with people in the same area and outside the local area); (5) trust (e.g. the extent to which people in the area can be trusted, being afraid to walk alone after dark); (6) attachment to neighbourhood (e.g. belief that neighbours are friendly, feeling part of the area); (7) tolerance (e.g. belief that everybody in the area should have equal rights, people in the area are tolerant of others not like them, respect for privacy); and (8) being able to rely on others for practical support (e.g. feeling comfortable asking neighbours to run errands for each other during illnesses) [17,18]. Many of the components of this framework would fit our definition of 'psychosocial', but again the list does not refer exclusively to risk factors that may affect health through psychosocial processes (for example, it includes practical rather than emotional support).

MacIntyre et al have advocated a broader framework to describe the pathways in which neighbourhoods may effect health [19]. Besides considering the physical environment, services and amenities, this framework also considers the 'socio-cultural' characteristics of a neighbourhood and its reputation, some of which may be regarded as relevant to psychosocial theories of health. Neighbourhood characteristics cover a range of risk factors including those associated with the political, economic, ethnic and religious history of a community, current norms and values, the degree of community integration, levels of crime, incivilities and other threats to personal safety, and networks of community support. Neighbourhood reputation includes how a local area is perceived by its residents, how it affects their self-esteem, who moves in and out of the area, and how the neighbourhood is perceived by service or amenity planners, providers and investors.

Siegrist and Marmot have defined the 'psychosocial environment' as the sociostructural range of opportunities that is available to an individual person to meet his or her needs of well being, productivity and positive self-experience [5]. They emphasise the importance of self-efficacy and self-esteem. A psychosocial environment conducive to self-efficacy enables the person to experience control in terms of successful agency. A psychosocial environment conducive to self-esteem enables the person to connect him- or herself with others in a way that strengthens feelings of belonging, approval and success (in contrast to feelings of being excluded or of not getting anywhere despite one's efforts). Although his work has largely focused on workplace health, Marmot has co-authored a paper from the Whitehall study of UK civil servants (Chandola et al) that presents some evidence suggesting that low control at home associated with excessive household and family demands may have a greater adverse effect on the health of women compared with men [20].

In the design of this review, we combined elements from this broader literature to develop a search strategy to identify studies of psychosocial risk factors in the community. We included those risk factors that appeared to us to fulfil the bridging role between socio-structural and psychological characteristics described above, but we reiterate the point made earlier that risk factors considered to be 'psychosocial' may potentially affect health through non-psychosocial pathways. The main sets of themes we have focused on are (a) autonomy and control, (b) involvement, participation and empowerment, (c) social capital, social cohesion, trust and belonging, (d) social support (including specific types of support: e.g. emotional), social networks and receiving positive feedback (e) social diversity and tolerance, (f) vulnerability, security or safety, and (g) demands, role conflicts or role imbalance.

We have identified and synthesised findings from systematic reviews that report data on any kind of health measure in association with any of the above psychosocial factors within a home or neighbourhood residential setting. The review includes a broad range of risk factors and health outcomes because the psychosocial epidemiological literature we have scoped is similarly varied. We do not suggest that empirical evidence is available to demonstrate how psychosocial processes explain associations between each of the specific risk factors and health outcomes identified in this review (in our discussion section we call for more evidence of this kind). We also note that whilst our initial search strategy was intended to include risk factors pertaining to all the themes referred to above, the output from the process has yielded evidence on a more limited range of factors, and so our findings do not address the full spectrum of those themes. This report summarises the evidence we identified, prioritising findings from the more robust reviews.

## Methods

### Inclusion criteria

We included all published and unpublished reports in all languages describing systematic reviews or meta-analyses, the health effects of psychosocial risk factors, or associations between health and psychosocial risk factors in residential settings. Health effects included social, psychological, and physical effects that could be measured on humans and health behaviours.

### Exclusion criteria

We excluded literature reviews that did not meet our minimal criteria (described below), descriptions of environmental or physical effects that did not include human responses to them, studies without health measures and studies reporting predicted but not observed health impacts of psychosocial risk factors.

### Search methods

We searched the following electronic databases from 1986 to date of search (at least October 2006): Centre for Reviews and Dissemination Database of Abstracts of Reviews of Effects (CRD DARE), NHS Economic Evaluation Database (NHS EED), Health Technology Assessment (HTA) Database, OVID Medline, ISI Web of Science, Social Science Citation Index, Science Citation Index, CSA Sociological Abstracts, CSA Social Services Abstracts, ASSIA: Applied Social Sciences Index and Abstracts (CSA), OVID Psychinfo, EBM Reviews – Cochrane Database of Systematic Reviews, EBM Reviews ACP Journal Club.

We used DARE's database-specific strategies to search for systematic reviews and meta-analyses [21]. Subject keywords were "(psychosocial or psycho-social or stress or involvement or empowerment or autonomy or contingency or participation or bridging social capital or control or choice or diversity or empowerment or esteem or hopelessness or insecurity or social network or social cohesion or social diversity or social support or vulnerability or security or safety or trust or demand or conflict or imbalance or allostasis or allostatic load) and (house or home or neighbourhood or neighbourhood or resident or town or city or community or inner-city or estate)."

We also searched personal collections, and other internet resources (including web of science citation searching), manually searched bibliographies and contacted experts.

### Evaluation of included reviews

One reviewer excluded obviously irrelevant documents. Two reviewers independently appraised papers using a checklist adapted for epidemiological reviews from two critical appraisal guides: CRD's DARE criteria for quality assessment of reviews [21] and a systematic review tool created by Oxman and Guyatt [22].

The checklist was based on seven criteria described in table 1 [see Additional file 1]. A quality index, based on these criteria, could range from 2 (numerous flaws) to 7 (minimal flaws). We required, as a minimum, that systematic reviews included a defined research question and an explicitly described search strategy. A distinction was made between higher and lower scoring systematic reviews: reviews that scored higher than 4 were placed in the 'higher' category. In recognition that the quality of a review is distinct from the quality of evidence included in a review, we distinguished between evidence obtained from longitudinal, cross-sectional and case control studies and reported the size of studies included in each review (when reported by the reviewers). Disagreements were resolved by the reviewers on a case by case basis.

### Data extraction

Information reported in each review on the number, design and findings of relevant primary studies were extracted, as well as data on environment, participants, psychosocial variables and health measures. Data on primary studies included in each review were extracted from the reviews rather than from the primary studies themselves.

When extracting data describing settings, we referred to the requirement that 'psychosocial' should be a bridging concept that links psychological factors to specific environments [14]. For example, if a review provided evidence on how a health outcome may be associated with social support (or lack of it) received from participants' spouses at home, we categorised the psychosocial environment as 'home.' If a review provided evidence on support from friends or neighbours outside the home, we categorised the psychosocial environment as 'community.' Often reviews provided evidence pertaining to both types of psychosocial environment.

Some reviews reported on a range of risk factors, not all of which met our operationalised definition of a 'bridging' psychosocial factor. In such cases, we only extracted and reported data relevant to psychosocial factors that met our inclusion criteria.

Data were abstracted by one reviewer (ME) and checked by another (ST). When more than one review reported on the same risk factors and health outcomes we prioritised findings from the more robust reviews.

## Results

Altogether 9638 titles and abstracts were screened by ME to exclude obviously irrelevant publications. 306 publications were obtained and considered for suitability. Of these, 62 reviews were considered to have met the subject inclusion criteria and were critically appraised. Thirty-one reviews met minimum critical appraisal criteria (scoring 2 or over [see Additional file 1] [23-53]). Most of the reviews reported on studies conducted in North America (particularly USA) and to a lesser degree northern Europe, although some evidence also emanated from the southern continents. All the reviews were English language except one (published in German [37]).

### Overview of included reviews

Most of the reviews included studies that measured more than one psychosocial factor, and many also measured multiple health outcomes [see Additional file 1]. The following domains of psychosocial risk factor were covered in the included reviews: social support, social networks, social capital, social cohesion, collective efficacy, participation in local organisations (often religious organisations), demands, support within familial and marital relationships, exposure to community violence or anti-social behaviour, exposure to discrimination, and stress related to acculturation to western society

Only 2 included reviews explored psychosocial factors that related exclusively to the home environment [33,53]. Of the remaining reviews, about half explored both home and community psychosocial environments, whilst the rest focused exclusively on communities. As the reviews tended to provide relatively few details on the settings of included studies, it is not possible to provide detailed definitions of what is meant by the term 'community' for each study, or even each review. Its meaning varied both in terms of scale and in terms of whether 'community' was being used to describe social networks, a geographical location (e.g. residential neighbourhood) or (more usually) a hybrid of the two.

### Findings from higher scoring reviews

Of the 31 reviews we identified, 11 scored > 4 out of 7 from the critical appraisal [23,25,32,33,37,38,40,43,49,50,53]. All but two [32,38] of these more robust reviews were published in the last five years. Even amongst the higher scoring reviews, reporting of numerical data and the methodological characteristics of primary studies was at times sketchy or absent.

Summarised findings from all 11 of these reviews have been ordered by psychosocial risk factor and are presented below and in table 2 [see Additional file 2]. Table 2 also summarises information on study designs and sample sizes. This is to help give readers some indication of the strength of evidence reported in each review: for example, methodological characteristics such as prospective longitudinal designs and large sample sizes may provide safeguards against some types of error. Evidence from single cross-sectional studies provides little or no indication of causal associations and such studies are often regarded as less robust in comparison to longitudinal studies (but we note that the appropriateness of a study design is partly determined by specific research questions and contexts, and that a well conducted cross-sectional study could potentially be more reliable than a poorly conducted longitudinal study). Three of the higher scoring reviews reported that only longitudinal studies were included [23,25,33], whilst a further 6 included longitudinal studies and studies with other designs (e.g. cross-sectional) [23,32,37,40,43,49].

#### Social support

Social support and social networks were not always clearly described or distinguished in the included reviews. Measures of support also varied: e.g. Kuper et al (who authored the most robust review on social support and coronary heart disease we identified) state that " [d]espite the interest in social support, there is little consensus on how it is measured, therefore variables ranging from 'high love and support from wife' to 'social network index' to 'social isolation' were included" [23]. Out of 21 prognostic studies of social support reviewed by Kuper et al, 10 were strongly supportive of an inverse association between social support and CHD whilst 4 were moderately supportive. No studies showed negative outcomes. Some large studies, including the largest, found little association [23].

Garssen identified evidence of a relationship between social support and disease progression amongst (mainly breast) cancer patients from 6 studies, as well as nine studies that found little or no association [25]. It should be noted that this review provided evidence on the course and progression of cancer, not its onset.

Smith et al reviewed 67 studies of social support (e.g. size of social network, availability of supportive people, informational or emotional support) and physical, psychological, and stress-related ill health [32]. Associations were usually positive but small in magnitude and the overall findings were inconclusive.

#### Support from a partner

It may be hypothesised that support from a spouse may benefit health but there are alternative explanations for positive associations between marriage and health (e.g. health selection hypotheses and the potentially harmful effects of bereavements and relationship breakdowns). A meta-analysis of marital status and mortality pooling 53 independent cohort studies, concluded that marriage was associated with lower mortality (RR = 0.94; 95% CI = 0.92 to 0.95) [33]. A review of risk factors associated with the emergence of bipolar disorder (BPD) found some evidence that living alone tends to be associated with an elevated risk for BPD compared with married or cohabiting persons [53].

#### Social support and participation

In a review of functional status decline in elderly people, Stuck et al found that 'low frequency of social contact,' 'participation in local activities' and 'greater frequency of emotional support from social networks' had positive associations with functional outcomes [38]. The reviewers rated the evidence on frequency of social contact as particularly robust [see Additional file 2]. Greater frequency of instrumental support was associated with increased risk of subsequent disability amongst older men.

Bernhardt et al identified three prospective longitudinal studies that found engagement in various forms of social and free time activities was associated with reduced risk of dementia amongst elderly populations, whilst reduced social contacts and living alone increased the risk [37]. One case control study also found that Alzheimer sufferers were less likely to have engaged in social activities, but two other case control studies found no evidence of an association.

Sellström and Bremberg identified some evidence that favourable neighbourhood *social climates *(including variables such as social support and control, crime rates, active voluntary associations, residential stability, neighbourhood cohesion, and collective efficacy) may benefit child health [40]. Children who already belong to advantaged groups seemed most likely to benefit. Some studies found little or no association between social climate and health [see Additional file 2]

#### Religious participation

Hackney and Sanders identified evidence of associations between religious participation, religiosity and mental health in a meta-analytical review [50]. However, forms of religiosity that focused on the social and behavioural aspects of religion (e.g. attendance at religious services, participation in church activities, etc) were found to be associated with greater psychological distress but higher life satisfaction [see Additional file 2].

Another meta-analysis examined the association between religiousness and depressive symptoms across 147 independent investigations and found that greater religiosity is mildly associated with fewer symptoms [49].

#### Family relationships and dysfunction

In a review of risk factors associated with the emergence of bi-polar disorder, Tsuchiya identified 4 studies measuring inconsistent evidence of associations between BPD and varying domains of family relationships and dysfunction [53].

#### Acculturation

Acculturation to western society (following immigration or 'westernisation' of developing countries) has been included in this meta-review because of the hypothesised role of social support and other psychosocial risk factors in mediating the health impacts of acculturation [54-56]. Nonwesterners have been characterised as having larger social networks and more social support than westerners, and as nonwesterners adapt to a western lifestyle, their level of social support decreases [57]. Alternative pathways connecting acculturation to ill health such as workplace psychosocial factors and health behaviour changes (diet, physical activity, etc.) have also been suggested [43].

Steffen et al conducted a meta-analysis of evidence on acculturation to western society and blood pressure [43]. Studies that evaluated systolic blood pressure found that acculturated individuals had an average of 4 mm Hg higher blood compared to less acculturated individuals, which the reviewers state to be similar to the effect sizes of known risk factors for high blood pressure such as body weight, level of physical activity and work stress. Effects did not appear to be related to body mass index or cholesterol (which the authors use as proxies for health behaviours such as diet and physical activity) and tended to decrease during the first few years of acculturation.

### Evidence from the other reviews

The higher scoring reviews referred to above provide evidence on most of the psychosocial risk factors identified in this review. However, quality appraisal should not be accepted uncritically [58]. For example, a robust review may have received a low score because the authors under-reported their methods, rather than because those methods were poor. Furthermore, high scores do not prevent reviews from becoming outdated over time.

We have therefore summarised the results of lower scoring reviews if (a) the psychosocial factors, health associations and settings they explore have not been covered by more robust reviews; (b) they present numerical data and/or details of included study designs to support their findings; and (c) they were published since 2000. These summaries are once again organised by psychosocial factor and presented below.

#### Social capital

Kawachi et al reviewed 31 studies of social capital and health in community settings [34]. The authors identified ecological and multi-level studies that provided inconsistent evidence of associations between community level social capital measures such as collective efficacy, trust, social control and social cohesion being associated with health outcomes such as general health, child health, and lower violent crime and homicide. The authors also identified some evidence suggesting negative associations: i.e. children in poor areas with mothers reporting low community attachment were associated with fewer behavioural and mental health problems.

#### Demands

In a review of gender differences in psychiatric morbidity among family care givers, Yee and Schulz found that female caregivers reported more caregiving demands, less likelihood obtaining practical support and more psychiatric symptoms compared to male caregivers, and to non-caregiving community samples [48].

#### Social support, cohesion and control

Rajaratnam et al identified evidence of associations between social resources (including measures of social control, cohesion, trust, reciprocity, collective efficacy, participation and community involvement) and infant birth weight, conduct disorder, child health and child maltreatment at the neighbourhood level [41]. However, the review focused on identifying measurement tools and did not report numerical data.

#### Exposure to community violence and discrimination

Research on the psychosocial impact of young people's exposure to community violence has been prompted by concern that exposure to violence (including witnessing) may be a stressful experience that requires psychological adaptation and that adverse psychological sequelae may result [59]. From a meta-analysis of 37 studies of unspecified designs (combined sample: n = 17322) Wilson et al found a positive correlation between exposure to community violence and psychological distress and reported a low-medium effect size (r = 0.25) for this relationship [47].

Similarly, there is growing scientific interest in examining the extent to which exposure to racial/ethnic discrimination may be considered as types of stressful life experience that can adversely affect health [60]. Williams et al have reviewed associations between racial/ethnic discrimination and various measures of mental and physical health [44]. The authors identified 53 studies, of which only 3 were longitudinal. All the studies were said to have at least one serious methodological flaw but the authors state (without supporting numerical data) that there was 'substantial consistency' of evidence of associations, 'especially among the methodologically strongest studies.'

#### Socio-marital support

A review of evidence of the association between attachment security and maternal mental health correlates found that a socio-marital support (a combined measure of support within and outside of marital relationships) was significantly related to parent-child attachment security [28]. However, evidence from prospective studies on the association between social support and marital support and postpartum depression produced inconsistent findings including positive, negative and inconclusive evidence of associations.

Finally, a review of risk factors predicting geriatric health identified 6 studies (2 longitudinal). Two studies found a positive association between social network size and health and wellbeing (including the largest longitudinal study: n = 6928; odds ratio = 1.76 (95% CI = 1.02 to 3.02)) [31]. The other longitudinal study and three cross-sectional studies found no conclusive evidence.

### Health inequalities

We examined the higher scoring reviews, and recent reviews presenting numerical outcomes and/or outcomes that prioritised more robust evidence, for any evidence that could shed light on how health inequalities may be associated with psychosocial risk factors [see Additional file 3]. Such evidence could potentially suggest strategies for tailoring interventions to reduce such inequalities. Nine reviews provided some evidence on interactions between social position and psychosocial factors but we identified no robust evidence describing how exposure to psychosocial risk factors was differentially distributed across population subgroups (e.g. categorised by gender, ethnicity, socioeconomic position, etc) [32,33,38,40,43,47-49].

We identified some evidence that living in high crime or violent neighbourhoods may adversely affect the health of people who belong to social groups that may also be disempowered or disadvantaged in other ways: such as ethnic minority groups, inner-city communities and women with low educational status [40,47]

Conversely, participants from more socially empowered groups may be in a better position to capitalise on the positive psychosocial characteristics of their communities. For example, it was generally the children of white mothers who appeared to benefit (in terms of higher birth weight) from being part of a community perceived to be high in social support (compared to mothers from ethnic minorities who also lived in supportive communities) [40].

Some studies found little evidence of differential effects, whilst findings on gender differentials did not fit simply into an 'advantaged vs. disadvantaged' interpretation. Women caregivers may be more susceptible than male caregivers to experiencing mental ill health associated with high demands [48], but older men's health has been found to have been negatively affected by instrumental support [38], and men may be more adversely affected by psychosocial factors linked to acculturation to western society compared to women [43].

The overall lack of robust evidence on health inequalities we identified may reflect the poor quality of some of the reviews, but it could also reflect a tendency sometimes visible in epidemiological studies and meta-analyses to seek a single overall outcome for all participants that controls for differences in gender, age, ethnicity etc, rather than to explore heterogeneity of outcomes amongst different sub-groups [9].

## Discussion

This meta-review (review of reviews) explores the evidence of psychosocial risk factors and their associations with population health in home or community settings. The evidence is of varying quality and focuses more on social support and networks than on other psychosocial factors. The review has identified evidence that appears to support hypothesised associations between specific psychosocial factors and health and evidence that does not demonstrate such associations.

### Summary of specific findings

The more robust reviews we identified do provide some stronger evidence from longitudinal studies that the quality of social support and size of social networks may be associated with lower risk of coronary heart disease and cancer (particularly breast cancer) [23,25].

There is also longitudinal evidence that social support (from spouses at home and from social networks in the wider community) and participation in local activities may be associated with better health amongst elderly populations [33,37,38].

We identified evidence that fewer social resources at a community level may be related to an increased likelihood of child maltreatment at home, whilst evidence from another review suggested that unsupportive or maladaptive family relationships at home may be associated with a higher risk of offspring developing bi-polar disorder in later life [41,53].

This meta-review also identified some less consistently robust evidence that children and young people from neighbourhood environments that are considered to have fewer psychosocial advantages (described differently in different reviews, but taking into account concepts such as social capital, social resources and social cohesion) may shoulder a disproportionately high burden of physical and psychological ill health across a range of measures. They may also experience family dysfunction, parenting problems and may be more likely to engage in risky health behaviours [41,47,53].

Evidence from North America suggests that members of ethnic minorities experiencing racial discrimination may be at greater risk of psychological distress, poorer physical health, and may be more likely to engage in unhealthy behaviours such as smoking and alcohol use [44]. Immigrant/migrant populations in western or westernized countries tend to experience high blood pressure, particularly during the initial period of acculturalisation, which has been attributed to stress-related psychosocial factors. Populations in less developed countries experiencing rapid industrialisation (or 'westernisation') may similarly be at greater risk of high blood pressure [43].

Overall, we identified some evidence that supports the view that favourable psychosocial environments are linked to better health, and some that demonstrates little or no association.

### Negative associations

The reviews we included identified little evidence of a favourable psychosocial environment being associated with unfavourable health outcomes, but there were some examples. Two reviews provide conflicting evidence about the relationship between religiosity, religious participation and mental health [49,50]. A review of social capital included evidence that children in poor areas with mothers reporting low community attachment were at less risk of developing behavioural and mental health problems [34]. Explanations for such findings may be speculated but the reviews provided little evidence upon which to test such speculations. As causal direction is not formally established, health selection may potentially explain some results (e.g. less healthy men may be more likely to receive greater levels of instrumental support). Some findings may also be interpreted in the light of research on the potentially negative effects of some forms of social capital: i.e. not all social relationships and not all forms of community engagement may necessarily be beneficial to all parties concerned [61-63]. Some of these psychosocial factors may have a 'downside' [63].

These exceptions aside, where associations have been identified, they are generally supportive of the view that favourable psychosocial environments go hand in hand with better population health and less risky health behaviours. However, the evidence that underpins our understanding of those associations is, judging from this meta-review, of variable quality and consistency.

## Limitations

The evidence base that this report has explored does not provide researchers and practitioners with a means of resolving their uncertainties on key issues such as the relative importance of psychosocial risk factors compared to other types of health determinant, the degree to which findings are generalisable across different communities, or the effects of attempting to modify exposure to these risk factors as part of a health improvement strategy. Many reviewed studies measured both self-reported health and self-reported psychosocial measures, raising the possibility that both sets of responses could potentially be influenced by participants' feelings of optimism or pessimism.

The included reviews also provide little evidence of the causal mechanisms that might explain specific health associations. Evidence of such mechanisms would have been particularly useful in refining our definition of 'psychosocial' and explaining inconsistent findings in the light of heterogeneous study populations, different socio-economic contexts and multi-level analysis (e.g. area-level variables may affect health through different processes than individual-level psychosocial factors).

Some of these limitations may be attributed in part to our approach to scoping this literature, but they also arise because clear quantifiable evidence capable of substantially resolving such uncertainty is not available: i.e. the literature itself has its limitations.

Our decision to focus on reviews, though considered necessary when investigating such a broad subject area, limits the comprehensiveness of our literature review and carries certain risks of bias and error. Publication bias may affect our findings on two levels. If publishers favour articles that report positive findings (as is commonly believed), the publication of literature reviews and the publication of primary studies included in those literature reviews may reflect this bias.

We have also been reliant on the authors of reviews accurately reporting the findings from the studies they have synthesised, just as those reviewers were themselves reliant upon the authors of primary studies maintaining high standards of reporting.

Two thirds of the included reviews were published within the last five years but six were over 10 years old. They cannot have included recent primary research. Some of the reviews did not appear to involve comprehensive literature searches. Furthermore, critical appraisals of the included studies were not always conducted and review findings did not always prioritise stronger evidence from methodologically robust studies over weaker evidence from less robust research. Because many of the reviews do little to shed light on the methodological quality of included studies, it is not possible to gauge the extent to which positive findings may reflect genuine associations or study bias.

### Research implications

The findings from this review, and some of the limitations we have identified, have implications for future systematic reviews, as well as for theoretical work and primary empirical research in the field of psychosocial epidemiology.

#### Implications for systematic reviews

Primary research in public health has been criticised for including a disproportionately low number of evaluations of the effectiveness of interventions (compared to, say, epidemiological evidence of disease prevalence and risk factors) [8,9]. In contrast, whilst primary research is predominantly descriptive, systematic reviews tend to focus on intervention evaluations rather than descriptive evidence (e.g. see the systematic reviews in the Cochrane Library [64].

This disparity is especially problematic to those interested in 'upstream' determinants of health and health inequalities such as housing, neighbourhoods and community environments. Robust evaluations of interventions affecting such determinants are difficult to conduct and are particularly rare [8,9]. Descriptive epidemiological evidence is more readily available. So, whilst we believe that it is essential that more primary research should be directed towards evaluating complex social interventions affecting upstream health determinants, we also believe that systematic reviews should be used to appraise relevant evidence that is currently available in this field: including descriptive evidence.

Systematic reviewers have tended not to do this, which has had an impact on this meta-review. Despite our relatively broad research question we found only 31 reviews and few of these scored highly in our appraisals. If epidemiological evidence is not being identified, appraised and synthesised robustly, this is an obvious cause for concern. We therefore recommend more systematic reviews designed to identify the best available evidence on upstream determinants of health including evidence of disease prevalence and risk factors, as well as intervention studies.

#### The need for better theory and definitions

The main theoretical problems we identified were the lack of evidence of causal pathways and a lack consistent definition of 'psychosocial.'

Regarding the former, we have identified a need for further research and theoretical development on mechanisms that could explain how specific psychosocial factors affect different health outcomes, and greater efforts should be made to design empirical studies to test such theories.

Regarding the defining of 'psychosocial,' even the most robust reviews we identified include some risk factors that appear to us to be 'psychological' rather than 'psychosocial': such as depression, anxiety, distress and type 'A' behaviour [23]. Whilst our own operationalised definition of 'psychosocial' is not intended to be in some way the 'last word' in defining this term, we do feel that to be of any value in its own right, the concept of 'psychosocial' must be distinguishable from (albeit related to) psychological factors and processes. We accept that our final list of risk factors may be contested and indeed we would welcome debate as part of a process of seeking greater consensus in defining this subject area. We also accept that some readers may choose to focus on the outcomes we reported that best fit their own view of what they mean by 'psychosocial.'

The problem of definitions was particularly acute because of this meta-review's focus on community settings outside the workplace. The task of assembling a list of risk factors that might be regarded as both 'psychosocial' and relevant to home and neighbourhood settings was not straightforward. This is because some of what are arguably the most influential psychosocial models (e.g. the demand control support model and effort-reward imbalance model) have been developed and tested in predominantly workplace setting [3,4,10]. Whilst the concepts of demand, control and support may 'translate' from workplaces to homes and neighbourhoods in the form of (for example) housework or informal caregiving demands, participation in local decision-making and autonomy within the home, or familial and neighbourly support, there remains the issue of whether other psychosocial factors and theories can be put forward that are perhaps more relevant to residential rather than employment settings.

#### Implications for empirical research

By far the most widely researched psychosocial factors we identified in this meta-review were social support and social networks. In contrast we found little evidence on empowerment, control or demands within home or community settings. This provides an interesting contrast with the literature on psychosocial factors in the workplace, in which control is often posited as the psychosocial factor with the strongest health effect [13].

The predominance of social support and networks in the research literature on population health does not seem to reflect any empirically founded assumptions that these are the most influential psychosocial factors. We have identified evidence that suggests participation, cohesion, (less) exposure to violence and discrimination, can all have health associations. We can make no conclusion that one factor is more important than another: only that some feature more prevalently in the research and review literature.

Hence, we recommend that more research be conducted on other psychosocial factors besides support and networks. The list of such factors is potentially long, so it may be useful to suggest a priority. We would emphasise the need for more research on control, autonomy and empowerment in both home and community settings. We suggest this partly because evidence from the workplace appears to underline the importance of control, and partly because much of the rhetoric surrounding neighbourhood and community improvement initiatives give prominent place to the importance of different forms of empowerment [13,39,65].

As there is good evidence to suggest that control in the home environment has different (greater) health benefits for women than men (who conversely seem to benefit more from control at work), it is also important to explore differential effects of psychosocial factors [20]. As psychosocial factors have been posited as important causes of health inequalities [66], it is essential that more comparative research (comparing gender, education, income, ethnicity, age and other relevant variables) explore the social patterning of psychosocial factors and health, and the mechanisms that underlie those patterns.

Finally, many of the studies identified in our included reviews were small and/or used cross-sectional designs and were therefore unable to conclusively demonstrate associations or direction of causality. As non-causal relations cannot be expected to form the basis of effective public health interventions, causality needs to be explored further [6]. Besides conducting longitudinal studies, evidence on causality can be obtained by evaluating the outcomes of interventions [67]. It goes without saying that future research should, if it is to significantly advance our understanding, be sufficiently robust. We recommend large, multi-site longitudinal studies, designed with control or comparison groups as appropriate.

#### Policy implications

The policy community has become increasingly interested in the health effects of psychosocial factors, but we would urge policy-makers to seek greater clarity with regards to both what is meant by the term psychosocial and what the mechanisms are by which psychosocial improvements are expected to affect health. This review has identified psychosocial factors that could potentially be encouraged (such as social support and participation) or discouraged (exposure to crime, violence or racism in the community) to improve people's health and well-being. The evidence base we identified is flawed but we would argue that limitations to the evidence base should not dissuade policy-makers from making financial, legal, educational, technical and human resources available to promote more cohesive and supportive, and less anti-social, communities. Such community improvements are desirable for reasons that extend beyond considerations of their health effects, and would provide further opportunities for more health-focused evaluations.

## Conclusion

This meta-review was intended to describe the evidence base connecting psychosocial factors to population health in home and community settings. It informs readers about the kinds of evidence that is available, where the evidence is thin, and in which directions psychosocial theory might be developed in future. It has reported on evidence of psychosocial factors contributing to health inequalities linked to multiple disadvantage, and psychosocial factors associated with health across populations.

It is time for researchers and policy-makers to make a more concerted effort to understand the complex relationships between psychosocial environments and health for different social groups in the community. Researchers and policy-makers also need to explore (and evaluate) how those environments might be modified to improve health and reduce health inequalities. Finally an effort is required to compare the potential benefits of modifying psychosocial environments with the effects of other forms of social and environmental change – including economic strategies and improvements to the physical environment. In this way psychosocial epidemiology can find its place within more general epidemiology, and policy-makers will have a better understanding of which kind of strategies will yield the greatest benefits to the health and wellbeing of the people they serve.

## Competing interests

The authors declare that they have no competing interests.

## Authors' contributions

ME, CT and MP planned the study. ME and ST collected and analysed the data. All the authors assisted in writing-up.

## Pre-publication history

The pre-publication history for this paper can be accessed here:



## Supplementary Material

Additional file 1 Table 1: Quality, psychosocial variables, participants and settings of the included reviews. This table summarises the 31 psychosocial risk factor reviews identified through the literature search. Click here for file

Additional file 2 Table 2: Summary of psychosocial reviews with quality appraisal score > 4. This table summarises the 11 higher scoring reviews that have been identified. Click here for file

Additional file 3 Table 3: Psychosocial factors and health inequalities: summary of reviews that compare findings for different population subgroups. This table summarises reviews presenting data on psychosocial risk factors and health inequalities. Click here for file
