# Supplementary material for: Psychosocial risk factors in home and community settings and their associations with population health and health inequalities: A systematic meta-review
Source: BMC Public Health. 2008 Jul 16;8:239. doi: 10.1186/1471-2458-8-239 (PMC2503975; doi:10.1186/1471-2458-8-239)
Supplement: Additional file 1 — Table 1: Quality, psychosocial variables, participants and settings of the included reviews. This table summarises the 31 psychosocial risk factor reviews identified through the literature search. [file 1471-2458-8-239-S1.doc]

Table 1: Quality, psychosocial variables, participants and settings of the included reviews

| **Appraisal Criteria** | **Kuper [23]** | **Eriksen [24]** | **Garssen [25]** | **Alloway[26]** | **Robertson et al[27]** | **Atkinson [28]** | **Freisthler [29]** | **Fratiglioni [30]** | **Depp [31]** | **Smith [32]** | **Manzoli [33]** |
| --- | --- | --- | --- | --- | --- | --- | --- | --- | --- | --- | --- |
| **Is there a well defined question?** | ++ | ++ | ++ | + | ++ | ++ | ++ | ++ | + | ++ | ++ |
| **Is there a defined search strategy?** | ++ | + | ++ |  | + | ++ | ++ | + | + | ++ | ++ |
| **Are inclusion / exclusion criteria stated?** | ++ | + | + |  | ++ | ++ | ++ | + | + | ++ | ++ |
| **Are study designs and number of studies clearly stated?** | ++ | + | ++ | + | + | + | + | ++ | + | + | + |
| **Have the primary studies been quality assessed?** | ++ |  | + | + | + |  |  | + | + | + | ++ |
| **Have the studies been appropriately synthesised?** | ++ |  | + | + | + | + |  |  | + | + | ++ |
| **Has more than one author been involved at each stage of the review process?** |  |  |  |  |  |  |  |  |  |  | + |
| **Psychosocial risk factors** | Social support, social networks | | | | | | | | | | |
| **Psychosocial environment** | Community and home | | | | | | | | Community | | Home |
| **Health Measures** | Coronary heart disease | | Cancer | Depression | Postpartum depression | Parent child attachment | Child maltreat-ment | Dementia | Physical and mental health and wellbeing and health behaviours | | Mortality |
| **Participants** | Adults, patients | Adults, immigrants | Adults, patients | Adults, students, parents | Mothers | Mothers and children | Parents | Elderly | | Adults | Elderly |

Quality Appraisal Key: ++ yes; + partial; blank cell = no

Table 1 continued

| **Appraisal Criteria** | **Kawachi et al [34]** | **Ellitot [35]** | **Anderson [36]** | **Bernhardt[37]** | **Stuck [38]** | **Laverack [39]** | **Sellstrom [40]** | **Rajaratnam[41]** | **Sampson[42]** | **Steffen et al[43]** |
| --- | --- | --- | --- | --- | --- | --- | --- | --- | --- | --- |
| **Is there a well defined question?** | ++ | ++ | ++ | ++ | ++ | ++ | ++ | ++ | ++ | ++ |
| **Is there a defined search strategy?** |  | + | + | + | ++ | ++ | + | + |  | ++ |
| **Are inclusion / exclusion criteria stated?** | + |  |  | ++ | ++ |  | ++ | ++ | ++ | ++ |
| **Are study designs and number of studies clearly stated?** | ++ | ++ | + | ++ | ++ |  | ++ | + | + | + |
| **Have the primary studies been quality assessed?** |  | ++ |  | + | ++ |  | ++ | + |  | + |
| **Have the studies been appropriately synthesised?** | + | + |  | ++ | + |  | ++ |  |  | + |
| **Has more than one author been involved at each stage of the review process?** |  | + |  |  | ++ |  |  |  |  | ++ |
| **Psychosocial risk factors** | Social capital  Social power and social support | | Social support, participation | | | | | Social cohesion, collective efficacy re-anti-social behaviour | | Acculturation to western society |
| **Psychosocial environment** | Community | Community and home | | | Community | | | | |  |
| **Health Measures** | Physical and mental health | Coronary heart disease | | Dementia | Physical, mental health, mortality | Health behaviour | Birth weight, injury and maltreatment, conduct disorder. | | | High blood pressure |
| **Participants** | Adults, adolescents | Women | Adults | Elderly | | Adults | Infants, children, adolescents | | | Adults, immigrants, ethnic minorities |

Quality Appraisal Key: ++ yes; + partial; blank cell = no

**Table 1 continued**

| **Appraisal Criteria** | Willaims [44] | **Ingoldsby [45]** | **Veneema [46]** | **Wilson[47]** | **Yee [48]** | **Smith [49]** | **Hackney [50]** | **Mahoney [51]** | Foxcroft [52] | **Tsuchiya[53]** |
| --- | --- | --- | --- | --- | --- | --- | --- | --- | --- | --- |
| **Is there a well defined question?** | ++ | ++ | ++ | ++ | ++ | ++ | ++ | ++ | ++ | ++ |
| **Is there a defined search strategy?** | + |  | + | ++ | ++ | ++ | ++ | ++ | ++ | ++ |
| **Are inclusion / exclusion criteria stated?** | ++ | ++ | + | ++ | + | + | ++ | + | + | ++ |
| **Are study designs and number of studies clearly stated?** | + | + | + | + | + | ++ | + | ++ | + | + |
| **Have the primary studies been quality assessed?** |  |  |  |  |  | + |  |  |  | + |
| **Have the studies been appropriately synthesised?** | ++ |  | + | + |  | ++ | ++ | + | + | + |
| **Has more than one author been involved at each stage of the review process?** |  |  |  |  |  | + |  |  |  |  |
| **Psychosocial risk factors** | Discrimination | Exposure to community violence or  anti-social behaviour. | | | Social support, demands | Religiosity and participation | | | Family structure, support and relationships | |
| **Psychosocial environment** | Community | | | | | Home and community | | | | Home |
| **Health Measures** | Mental health, health behaviours | Conduct disorder, mental health, intellectual development | | | Mental health | Mental health | | Conduct disorder, mental health, intellectual development | Alcohol | Bi-polar disorder |
| **Participants** | Ethnic minorities | Adolescents | | | Adults | Adults, children, adolescents | | | Adolescents | Adults |

Key: ++ yes; + partial; blank cell = no
