# Supplementary material for: Psychosocial risk factors in home and community settings and their associations with population health and health inequalities: A systematic meta-review
Source: BMC Public Health. 2008 Jul 16;8:239. doi: 10.1186/1471-2458-8-239 (PMC2503975; doi:10.1186/1471-2458-8-239)
Supplement: Additional file 2 — Table 2: Summary of psychosocial reviews with quality appraisal score > 4. This table summarises the 11 higher scoring reviews that have been identified. [file 1471-2458-8-239-S2.doc]

**Table 2: Summary of psychosocial reviews with quality appraisal score >4**

| **Review First Author** | **Main Settings and Participants** | **Number* and Size (Smallest and Largest Sample Size) of Included Studies.** | **Findings on Associations Between Psychosocial Factors and Health** | **Summary Findings** |
| --- | --- | --- | --- | --- |
| **Kuper (2002) [23]** | Community and home settings.  Adults table – patients?.  North America, Europe. | No. of studies = 30 (9 etiological, 21 longitudinal prognostic studies)  Sample range: n=98, n=32624 | Out of 21 prognostic studies of social support, 10 were “strongly supportive” of an inverse association between social support and CHD (relative risks ≥2.00) whilst 4 were moderately supportive (relative risk ≥1.50 and <2.00). No studies showed negative outcomes. Some large studies, including the largest, found little association. | Some evidence of association:   - Social support/networks and reduced risk of coronary heart disease |
| **Garssen (2004) [25]** | Community and home settings.  Adults, patients.  North America. | No. of studies = 15 (all longitudinal).  Sample range: Not stated | 6 studies found a relationship between social support and disease progression. 9 studies found little or no conclusive evidence of an association between social support and cancer. This review reported little numerical data. In one study of 224 women with newly diagnosed breast cancer, the survival rate in women without a confidant was 56% compared to 72% for women who had had at least one type of confidant. | Some evidence of association:   - Social support and reduced risk of cancer (particularly breast cancer) |
| **Smith (1994) [32]** | Community setting  Adults.  Countries studied not reported. | No. of studies = 67 (24 longitudinal).  Sample range: n=11, n=3725. | Associations were usually positive but small in magnitude (weighted mean effect sizes ranging from -0.02 to 0.22). These effect sizes were the average of the reported correlations between each type of social support and health outcome transformed to consider sample size. Effect size results were equivalent when comparing studies grouped by gender of participants, study design and study quality. | Little or insufficient evidence of association   - Social support and physical/mental health |
| **Manzoli (2007) [33]** | Home setting.  Elderly people.  Europe and North America. | No. of studies = 53 (all longitudinal studies).  Sample range: n=206, n=93685. | Compared to married individuals, the relative risk of death for widowed participants was 1.11 (95% CI = 1.08 to 1.14); for divorced/separated individuals it was 1.16 (95% CI = 1.09 to 1.23); and for participants who never married it was 1.11 (95% CI = 1.07 to 1.15). A more conservative analysis of the data still found marriage to be significantly protective, although the effect size was reduced (RR = 0.94; 95% CI = 0.92 to 0.95). | Evidence of association   - Marital status and lower mortality |
| **Stuck (1999) [38]** | Community settings.  Elderly adults.  North America, Europe | No. of studies 21 (all longitudinal)  Sample range: n=91, n=6981 | The reviewers found strong evidence that ‘low frequency of social contact’ was associated with functional status decline (strong evidence defined as at least two appropriate analyses from separate databases (excluding people with functional status impairment at baseline) reporting a significant (p<0.005) increased risk of functional status decline in people having the risk factor at baseline, as compared to people without). They identified less robust evidence that participation in local activities and frequency of emotional support from social networks were associated with functional status decline. Instrumental support (e.g. provision of material aid or behavioral assistance) was found to have inconsistent and (for men) even harmful associations. Numerical data not reported. | Some evidence of association:   - Participation in local activities and reduced risk of functional status decline - Frequency of social contacts and reduced risk of functional status decline - Emotional support and reduced risk of functional status decline   Evidence of harmful association or little association   - Instrumental support and functional status decline |
| **Bernhardt (2002) [37]** | Community and home settings.  Elderly adults.  North America, Europe | No. of studies = 7 (4 longitudinal, 3 case control)  Sample range: n=90, n=4615 | In one longitudinal study, regular participation in social and free time activities was associated with significantly reduced risks of dementia (e.g. travel: RR = 0.48, 95% CI = 0.24 to 0.94; crafts/DIY: RR = 0.46, 95% CI = 0.26 to 0.85; gardening: RR = 0.53, 95% CI = 0.28 to 0.99). Another study found greater dementia risk (RR = 1.91, 95% CI = 1.12 to 3.25) and greater Alzheimer risk (RR = 2.68, 95% CI = 1.49 to 4.81) for unmarried people. A third study found that living alone and having no close social contacts increased the risk of dementia (RR=1.5, 95% CI = 1.0 to 2.1 and RR = 1.5, 95% CI = 1.0 to 2.4 respectively).  One case control study (n = 358) found Alzheimer sufferers were less likely to have participated in community activities than control group. Two case control studies (n = 120 and 90) found little or no associations. | Some evidence of association:   - Participation and reduced risk of dementia - Social contacts and reduced risk of dementia |
| **Sellström (2006) [40]** | Community setting.  Infants, children, adolescents.  North America, Europe. | No. of studies = 7 (3 longitudinal).  Sample range: n=400, n=101662. | In one cohort study, social support explained 15% of the variation in birth weight between neighbourhoods. In another, decreases in reciprocal exchange and participation in voluntary associations were significantly related to low birth weight (OR = 0.96). In neighbourhoods characterised by high criminality, a third cohort study found that the risk that women will give birth to low-birth-weight infants increased by 15% amongst women already experiencing individual disadvantage (defined by low education).[40]  Four cross-sectional studies found insufficient evidence that social climate effects childhood behavioural problems or the incidence of child maltreatment. | Some evidence of association   - Social support and higher birth weight - Reciprocity/participation and birth weight   Evidence of harmful association   - High crime rate and low birthweight |
| **Hackney (2003) [50]** | Home and community setting.  Adults.  North America | No. of studies = 35  Sample range not stated | Meta-analysis found an overall positive relationship between religiosity and mental health ( r= 0.10, 95% CI = 0.10 to 0.11, P<0.0001); religiosity related to less psychological distress (r = 0.02, 95% CI = 0.01 to 0.03, P<0.0001). Forms of religiosity that focused on the social and behavioural aspects of religion (e.g. attendance at religious services, participation in church activities, etc) were coded by the reviewers as ‘institutional religion’. Institutional religion was found to be associated with greater psychological distress (r = -0.03, 95% CI = -0.05 to -0.02, P<0.001) but also related to high life satisfaction (r = 0.10, 95% CI = 0.08 to 0.11, P<0.0001). | Some evidence of association:   - Religiosity and reduced risk of psychological distress   Some evidence of harmful association:   - Religious participation and psychological distress |
| **Smith (2003) [49]** | Community setting.  Adults (particularly students).  North America (some from Europe and other continents). | No. of studies = 147 (15 longitudinal).  Sample range: n=17, n=12007. | Across all studies, the correlation between religiousness and depressive symptoms was 0.096 (SE =0.0009, 95% CI = -0.11 to -0.08, P < 0.000001). The authors suggest that greater religiosity is mildly associated with fewer symptoms, but emphasise the heterogeneity of findings. Of 140 nonzero effect sizes (ranging from -0.54 to 0.24), 113 (81 %) supported the association whilst 27 (19%) found evidence of harmful associations. | Evidence of beneficial and harmful associations   - Religiousness and depression |
| **Tsuchiya (2003) [53]** | Home setting.  Adults, offspring  North America (some studies Europe) | No. of studies = 11  Sample range: n=19, n=5877 | Two small (n = 19 and 25) studies investigating child–parent relationships found that a dysfunctional relationship with parents during childhood and adolescence was associated with an increased risk for bi-polar disorder. One study (n=19) suggested that marital discord between parents was associated with an increased risk for BPD amongst offspring. A larger study (n = 5877) found some evidence of association between a father’s aggression and bi-polar disorder but not a mother’s aggression; however, the association disappeared after adjusting for subjects’ psychiatric comorbidity. Another smaller study did not support the association (n = 21 patient sample). Five of seven studies measuring marital status found that single persons tended to be associated with an elevated risk for BPD compared with married or cohabiting persons. Numerical data were not reported in this review. | Some evidence of harmful association:   - Dysfunctional family relationships and bi-polar disorder - Living alone and bi-polar disorder |
| **Steffen (2006) [43]** | Community settings.  Adults, immigrants, ethnic groups.  Africa, North America, South America, Europe, Asia | No. of studies = 125 (8 longitudinal)  Sample range: n=20, n=48817 | From 124 studies measuring systolic blood pressure, the random-effects weighted average effect size was d = 0.28 (SE = 0.023, 95% CI = 0.24 to 0.33, P < 0.000001), corresponding to an average difference of 4 mm Hg higher systolic blood pressure. Across the 114 studies that evaluated diastolic blood pressure, the random effects weighted average effect size was d = 0.30 (SE = 0.025, 95% CI = 0.25 to 0.35, P < 0.000001), corresponding to an average difference of 3 mm Hg higher diastolic blood pressure between acculturated versus non-acculturated samples. Effect sizes ranged from -0.75 to 1.83 for systolic blood pressure and from -0.44 to 1.53 for diastolic blood pressure, with the heterogeneity across studies being statistically significant for both evaluations of systolic blood pressure (Q(123) = 2551, P < 0.001) and diastolic blood pressure (Q(113) = 2415, P < 0.001), suggesting that systematic effect size variability was unaccounted for. | Some evidence of harmful association:   - Acculturation to western society and high blood pressure |

*Number of studies relates to the number of studies in each review that investigate psychosocial factors and their associations with health in home or community settings, as defined in our inclusion criteria
