# Supplementary material for: Psychosocial risk factors in home and community settings and their associations with population health and health inequalities: A systematic meta-review
Source: BMC Public Health. 2008 Jul 16;8:239. doi: 10.1186/1471-2458-8-239 (PMC2503975; doi:10.1186/1471-2458-8-239)
Supplement: Additional file 3 — Table 3: Psychosocial factors and health inequalities: summary of reviews that compare findings for different population subgroups. This table summarises reviews presenting data on psychosocial risk factors and health inequalities. [file 1471-2458-8-239-S3.doc]

**Table 3: Psychosocial factors and health inequalities: summary of reviews that compare findings for different population subgroups**

| **AUTHOR/DATE*** | **Smith (1994)[32]** | **Sellström (2006)[40]** | **Stuck (1999)[38]** | **Manzoli (1994)[33]** | **Yee (2000)[48]** | **Wilson (2003)[47]** | **Sellström (2006)[40]** | **Steffen (2006)[43]** | **Smith (2003)[49]** |
| --- | --- | --- | --- | --- | --- | --- | --- | --- | --- |
| **PSYCHOSOCIAL RISK FACTOR** | **Social support** | **Social support** | **Instrumental support** | **Marital status** | **Demands (informal caregiving)** | **Exposure to community violence** | **Exposure to high crime rate** | **Acculturation** | **Religiosity** |
| **HEALTH OUTCOME** | **General Health** | **High birth weight** | **Functional status decline** | **Mortality** | **Mental ill health** | **Mental ill health** | **Low birth weight** | **High blood pressure** | **Depression** |
| **GENDER** | No significant difference |  | Negative association for elderly men | No significant difference | Stronger association for mothers | No significant difference |  | Stronger association for men | No significant difference |
| **AGE** |  |  |  |  |  | No significant difference |  | No significant difference | Stronger association for elderly (weak evidence) |
| **ETHNICITY** |  | Stronger association for white mothers |  |  |  | Stronger association amongst black participants |  |  | No significant difference |
| **EDUCATIONAL STATUS** |  |  |  |  |  |  | Stronger association for less educated women |  |  |
| **GEOGRAPHICAL REGION OR COUNTRY** |  |  |  | Slight variance between northern and southern continents |  | No significant difference |  |  |  |
| **INNER-CITY, SUBURBAN OR RURAL** |  |  |  |  |  | Stronger association amongst inner-city participants |  |  |  |

*****Only reviews that reported data on differential health effects (or associations) of psychosocial risk factors on population subgroups (defined by gender, age, ethnicity, educational status, place and space) are featured in this table.

Note: blank cells refer to absences of evidence on differential outcomes; shaded cells refer to evidence of differential outcomes identified in this meta-review.
